# Educ@dom: comparative study of the telemonitoring of patients with type 2 diabetes versus standard monitoring—study protocol for a randomized controlled study

**DOI:** 10.1186/s13098-017-0252-y

**Published:** 2017-07-11

**Authors:** Marie-Christine Turnin, Solène Schirr-Bonnans, Jacques Martini, Jean-Christophe Buisson, Soumia Taoui, Marie-Christine Chauchard, Nadège Costa, Benoît Lepage, Laurent Molinier, Hélène Hanaire

**Affiliations:** 1 Department of Diabetology, Metabolic and Nutrition Diseases, University Hospital of Toulouse, 1 Avenue Jean Poulhès, TSA 50032, 31059 Toulouse Cedex 9, France; 2 DIAMIP Network, Toulouse, France; 3 ENSEEIHT, Toulouse, France; 4 Medical Information Department, Toulouse University Hospital, Toulouse, France; 5 Department of Epidemiology, Toulouse University Hospital, Toulouse, France

## Abstract

**Background:**

The global prevalence of type 2 diabetes is considerable. To avoid or delay its chronic complications, patients with type 2 diabetes should improve blood glucose management by adapting their life style. This involves changing the way in which diabetes is controlled. We believe that, thanks to technological innovations in connected health-monitoring devices, the telemonitoring of type 2 diabetes patients using therapeutic educational tools is likely to help them adapt their treatment and lifestyle habits, and therefore improve blood glucose management.

**Methods:**

This is a multicenter, randomized, controlled, prospective study. The primary objective is to compare the efficacy of telemonitoring to standard monitoring in terms of changes in glycated hemoglobin levels (HbA1c) after a 1 year follow-up period. The secondary objectives are clinical (changes in knowledge, physical activity, weight, etc.) and medical-economic. 282 patients are required (141 patients in each group) to satisfy the primary objective. For patients in the intervention group, the device will be given to them for 1 year and then withdrawn during the second year of follow-up.

**Conclusions:**

The anticipated benefits of this research are an improvement in blood glucose management in patients with type 2 diabetes by improving their lifestyle whilst rationalizing recourse to consultations in order to reduce the incidence of complications and cost in the long term. If the results of this study show that management of type 2 diabetes by tele monitoring is clinically effective and economical, this device could then be made available to a larger diabetic patient cohort.

## Background

According to the World Health Organization (WHO), 422 million people suffer from diabetes world-wide. This qualifies as a genuine pandemic since progression is considerable. Thus the WHO is forecasting that 622 million people will be suffering from diabetes between now and 2040 [1]. Over 90% of these people present type 2 diabetes. The severity of diabetes, from both a human and economic perspective, is the onset of chronic complications associated with the disease. To avoid or delay their onset as much as possible, patients with diabetes must improve the way in which they manage their blood glucose levels. Type 2 diabetes patients can be treated in different ways, depending on disease advance, ranging from a change in lifestyle alone, oral treatments or multiple insulin injections, or a combination of all options. The French Health Authorities (HAS) continue to advocate the following first-line therapy: a change in lifestyle with a change in diet and regular physical exercise for improved blood glucose management together with weight loss, if applicable [2, 3]. These measures must be maintained long-term even when combined with medication. Numerous studies have shown that a change in lifestyle can improve patients’ blood glucose levels but long-term results are difficult to maintain [4–6]. Thanks to technological innovation, health-related devices can now be used to monitor patients remotely in their own homes and play a greater role in daily routines by helping patients to improve their lifestyle and adjust their treatment [7–12].

We want to compare telemonitoring management incorporating therapeutic education tools for type 2 diabetes with standard monitoring. We believe that this telemonitoring management approach will improve patients’ blood glucose levels, lifestyle and quality of life whilst rationalizing recourse to consultations, thus helping to reduce the incidence of complications and long-term costs.

### Study objectives

The main objective is to compare the efficacy of telemonitoring versus standard monitoring in terms of changes in glycated hemoglobin (HbA1c) levels after a follow-up period of 1 year. Secondary objectives are changes in knowledge (evaluated using questionnaires focusing on dietetics and physical activity—GPAQ), hygiene and dietary approaches (3-day dietary questionnaires including 1 day at the weekend), quality of life (EQ 5D and DQOL questionnaires), clinical (weight, waist circumference) and laboratory parameters (lipid and mean glycemic fractions). These parameters are evaluated at baseline and after 1 year of follow-up. Patient and care satisfaction is also assessed after 1 year with continued efficacy (HbA1c) at 2 years. A medical-economic evaluation is also carried out and assessed after 1 and 2 years, respectively (cost-efficacy ratio, Markovian modelling for the longer term forecasting of medical and economic consequences).

Secondary objectives, specifically for patients in the intervention group are combined with the use of connected devices. They are defined to describe changes; in diet, in nutritional knowledge, in the extent to which physical exercise is carried out, in weight and body composition, in mean blood glucose levels and patient acceptability of the device.

### Study design

A multicenter, open-label, randomized controlled trial in patients has been carried out with type 2 diabetes patients, comparing an intervention group with the telemonitoring device under study versus a control group based on standard monitoring.

**Fig. 1:**
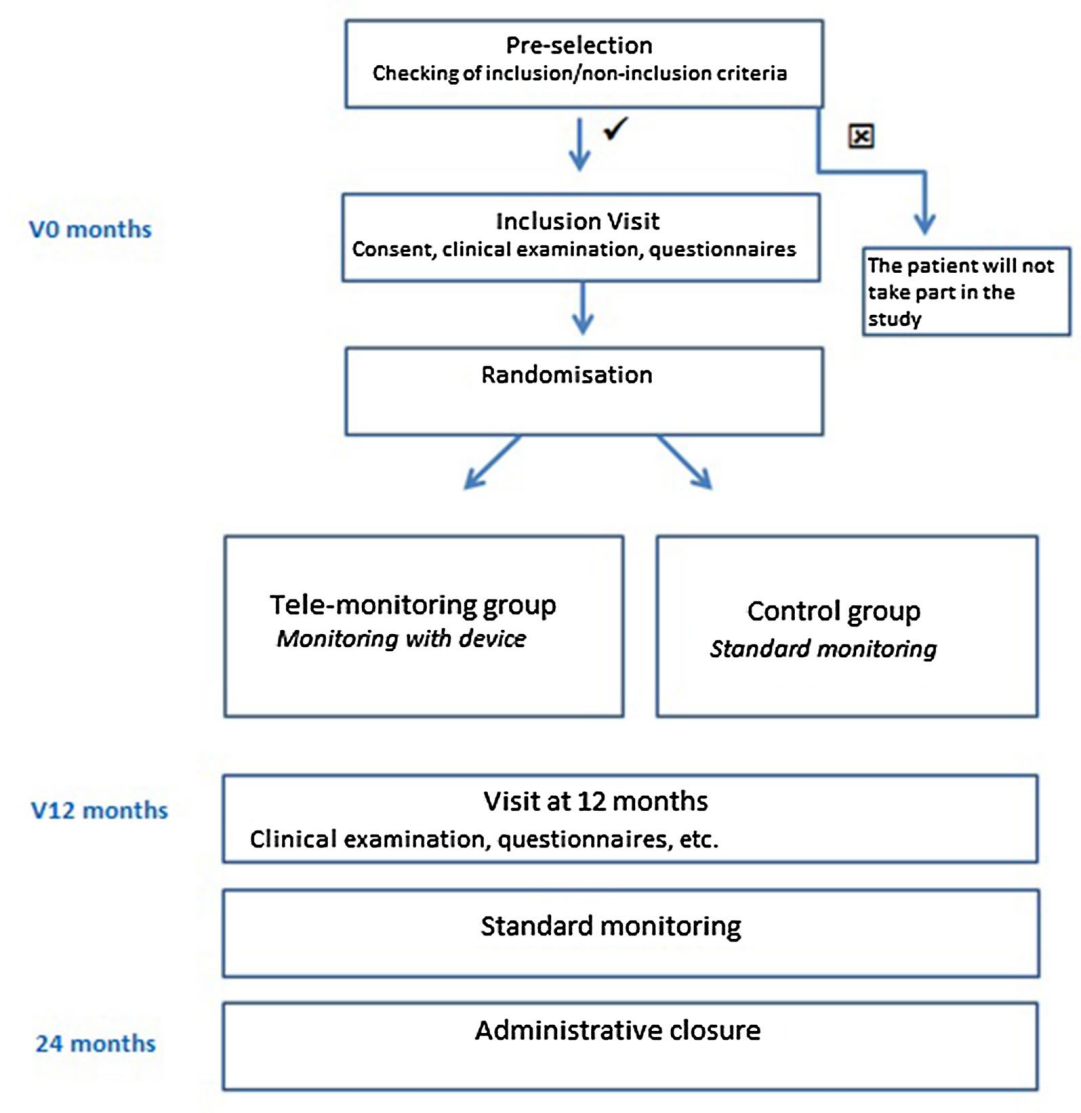
Study design and schedule. Comparative, randomized, open, multicenter intervention trial with parallel-group evaluating a telemonitoring program for patients with type 2 diabetes, whose glycemic control can be improved, compared to a traditional optimized care. Patients are recruited during therapeutic educational sessions or appointments by general practitioners or diabetologists. If patients accept to participate to the study and sign up the protocol consent, they are randomized into two groups: one arm is trained to the telemonitoring device before receiving it at home, and the other arm keeps usual care. During 12 months, the intervention group uses the device, and health teams use the web platform to follow the measured parameters and to make appropriate decisions about health care of their patient. Biomedical and economic data are collected along the study to assess medical and cost impact of the device between the two arms

The study procedure is summarized in Fig. 1.

## Methods

### Methods: participants, intervention and outcomes

#### Study setting

This study includes 16 patient recruitment investigating centers located throughout France: 5 university hospitals, one non-university medical center, a diabetology treatment network comprising 8 public and private health establishments and 2 independent physicians.

#### Inclusion and non-inclusion criteria

The patient inclusion and non-inclusion criteria are presented in Table 1.

**Table 1:** Patient’s inclusion and non-inclusion criteria

Inclusion criteria
Patient with type 2 diabetes
Patient over 18 years of age
Presenting blood glucose profile characterised by HbA1c levels ranging from >6.5 to 10%
With and without insulin
Having an internet connection at home
Agreeing to the training methods and to hire and use the device
Belonging to a social security system
Having given his/her informed consent and having signed the consent form
Non-inclusion criteria
Subject with reduced mobility
Subject living in an institution
Women wishing to have a baby, pregnancy, breast-feeding
Person participating in another clinical trial
Person placed under a legal protection system
Serious disease of recent onset (<3 months) or decompensated disorder likely to affect the patient’s blood glucose management in the long term
Confirmed haemoglobinopathy
Visual, intellectual or physical impairment
Inability to understand all or some of the software information
Retinal status not permitting optimisation of blood glucose management
Confirmed severe renal impairment (creatinine clearance <30 ml/min)
Person with severe dietary disorders
Person having undergone or about to undergo bariatric surgery
Person with an implanted electronic medical device

#### Intervention

Subjects are recruited from several investigating centers. After obtaining an informed consent, clinical examination is performed by the physician and questionnaires are collected. Patients are randomized into two groups: one arm is trained by the research staff on the telemonitoring device before receiving it at home through a demonstration device to familiarize with the connected objects. Instructions for use and the phone number of the project team are given to patients. Within 15 days a specialist in Health equipment, previously formed, comes in the patient home to install the device and explain to them the operation if necessary.

A hotline managed by our service provider is also available to patients to prevent them from experiencing technical or operational issues.

During 12 months, the intervention group uses the device, and health teams use the web service to follow the measured parameters and to make appropriate decisions about health care of their patients. Biomedical and economic data are collected along the study to assess medical and cost impact of the device between the two arms.

The other arm keeps usual care (Fig. 1).

#### Telemonitoring device (Fig. 2)

Tele-monitoring device available for use by type 2 diabetes patients in their own homes includes three educational software applications [13–15] focusing on a balanced diet and physical activity, available on tablets, and biomedical data sensors (scales with impedancemetry, actimeter, blood glucose monitor).

**Fig. 2:**
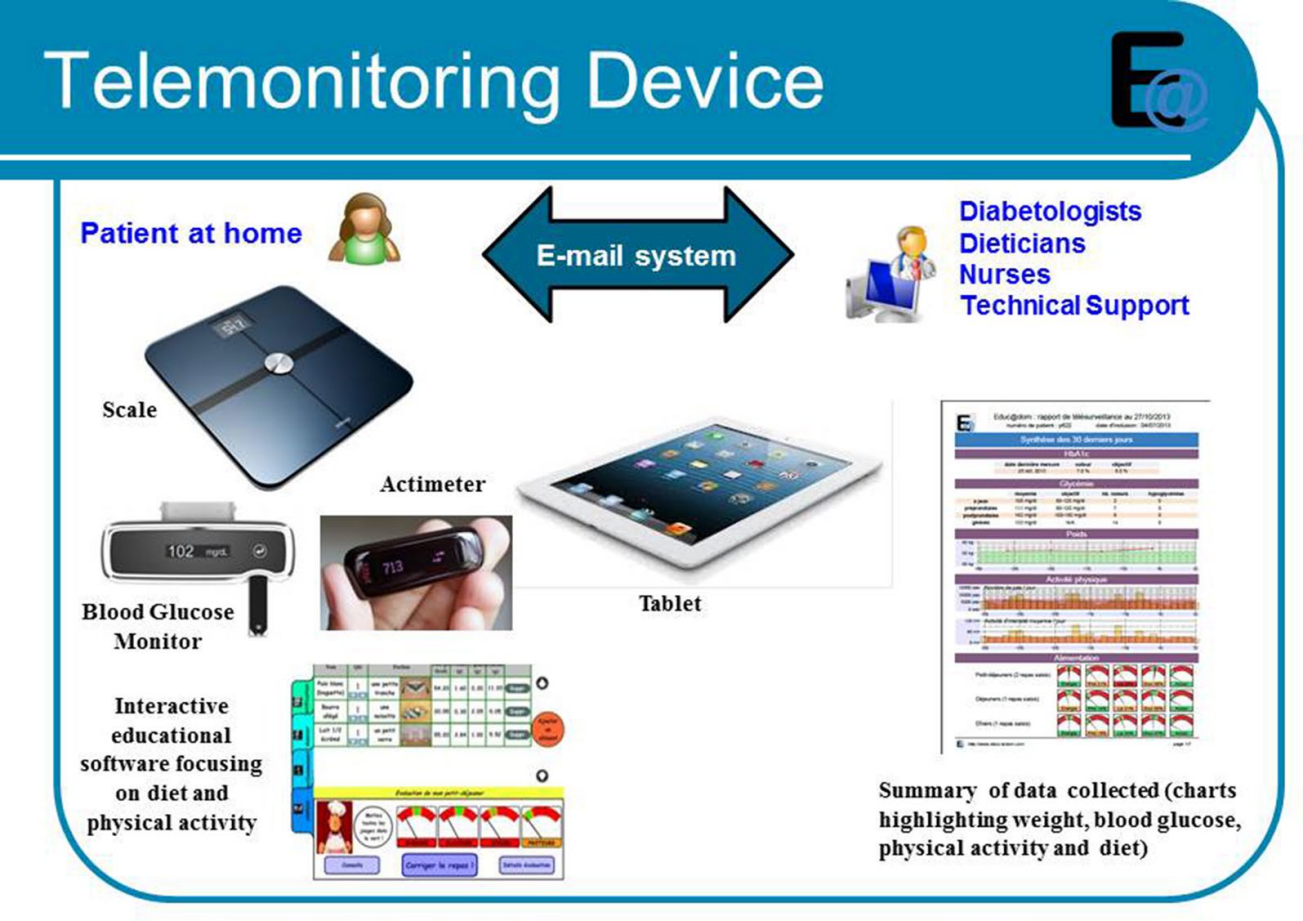
Telemonitoring devices available for the patient at home. A telemonitoring device with educational tools is given to people with type 2 diabetes at their home. It is composed of three software applications available on a tablet to improve eating habits, a self-monitoring blood glucose device, a connected scale to measure weight and fat mass, and a pedometer to evaluate physical activity. Secured web services are used to send and store data and to deliver a synthesis to patients and health professionals

##### Educational software

Patients in the telemonitoring arm will have a tablet at home which they can use to access 3 interactive educational software applications: Nutri-Educ, Nutri-Kiosk and Acti-Kiosk.

**Fig. 3:**
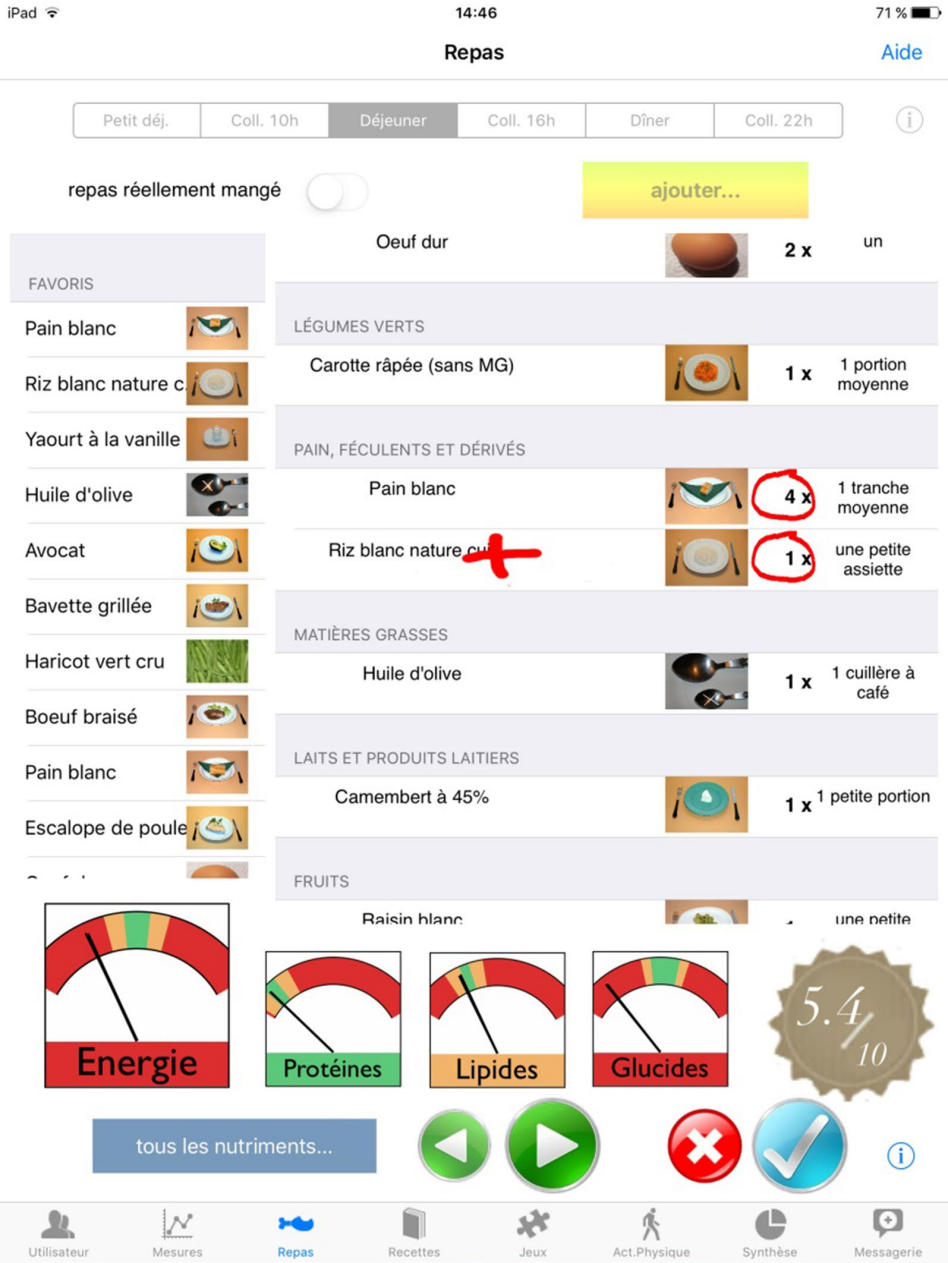
An example of meal with a proposition of correction to improve the nutritional balance. Nutri-Educ is an interactive, educational, nutrition software application that provides personalized help in the composition of meals. Each user has a personalized folder that contains some of its physiopathological characteristics. It was developed by the department of Diabetology of the University Hospital of Toulouse in collaboration with a computer engineer. It has several features: the system evaluates the energy needs of the subjects by a set of rules, taking into account age, sex, size, physical activity. The caloric goal is then adapted according to the BMI of the subject. It helps the user to balance his meals with personalized advice. It takes into account the daily fractionation of the feed. The user enters the foods that make up his/her meal with photos to choose the type of food and its portion. Nutri-Educ makes a diagnosis of the meal calorically and on the carbohydrate-lipid-protein distribution according to the international recommendations. It mentions the lack of fiber, calcium… and then indicates several food combinations involved in the imbalance of the meal. It is able to offer balanced meal solutions from the foods seized by the user while trying to respect the choices of the subject as well as possible

Nutri-Educ is a personalized nutritional educational software application that meets international clinical trial recommendations [16]. It allows patients for whom a profile and nutritional requirement record has been created to enter their meals (in the form of photos with various portions of food), to establish whether or not their meals are balanced and to obtain one or more suggestions for improving the nutritional balance (Fig. 3). These meals can be recorded and used in the patient telemonitoring process. Based on feedback from the teaching units in our department of Diabetology, we assessed that asking for the description of six meals per month is the most feasible.

Patients are asked to enter 2 breakfasts, 2 lunches and 2 dinners per month as a minimum requirement. Nutri-Kiosk is a series of quizzes which test patients’ nutritional knowledge. EDUC@DOM patients are advised to use it at least once a month.

Acti-Kiosk is a software application that allows patients to assess their physical activity/sedentary behavior. It gives tips on how to start up a physical exercise program (warm-ups, stretching, adjusting the bicycle saddle, etc.) in the form of videos.

##### Connected health devices

In addition to these education software applications, connected health devices form part of the telemonitoring system: connected blood glucose monitor to record blood glucose readings taken by patients, a connected scale with impedancemetry to monitor weight (with fat mass and lean mass) and an actimeter to monitor physical activity (number of steps taken, climbing stairs or hills, intensity of the activity). Some of the connected devices such as the scales do not require any intervention on the part of the patient to send the data, whilst others require data to be downloaded (blood glucose monitor) or the device has to be positioned next to the tablet (actimeter). Patients are asked to submit their data once a week.

##### The web telemonitoring platform

**Fig. 4:**
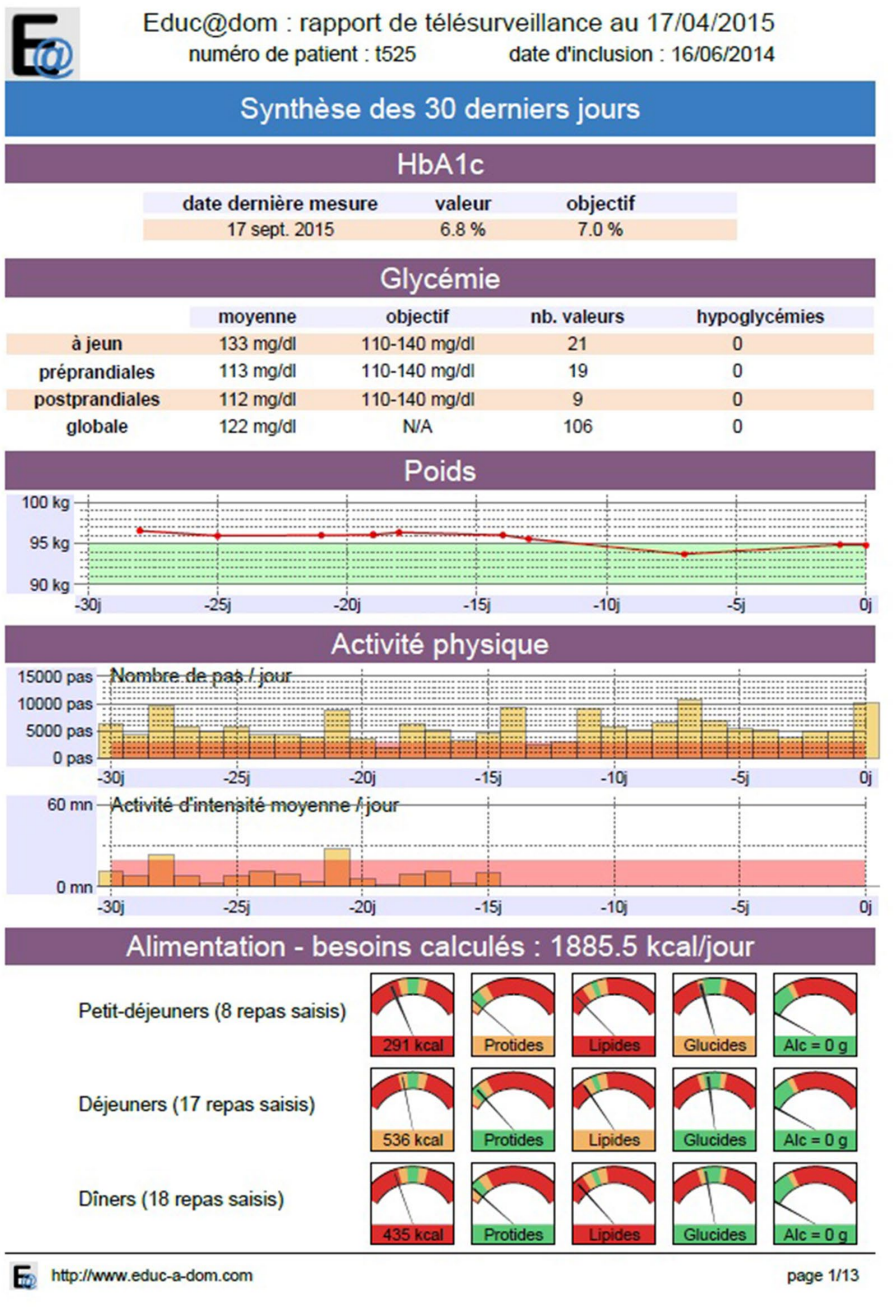
Remote monitoring report summarizes the important parameters of diabetes. A module on the tablet will synthesize all of this information and provide an assessment to the patient at the time of his choice in the form of tables and graphs: weight curve, evolution of pre- and postprandial glycemic averages, Physical activity (number of steps recorded by the actimeter, number of stages assembled, time of physical inactivity), evolution of dietary behavior (evolution of caloric intake and quality of nutrients (calories, carbohydrates, lipids, proteins…). This module will send technical alerts to the project team if the tools are not used. A module available on a secure server will store and synthesize this data and make it available to caregivers (general practitioners, diabetologists, and dieticians) in monthly synthesis form. They will be able to see this synthesis which will enable them to refocus their objectives relative to patients in a personalized way

All of these data are sent to a secure web platform. A software application translates these data in the form of graphs and charts to produce a summary that can be accessed by the doctor and by the patient via the tablet, on request. This summary (Fig. 4) includes an initial display of data over the last 30 days (weight, number of steps taken, intensity of the physical activity, targets in the form of benchmarks for meals, blood glucose diary) with a month-by-month average of the various follow-up parameters. All of the meals entered by the patients can also be accessed.

Using this platform, the investigators can also access the patient record and can amend the targets initially predicted at the inclusion visit that patients have to reach over time: number of steps, blood glucose targets or weight. These objectives are also viewable by the patient in the summary at any time.

In the intervention group, patients are remotely monitored for the first year and are then followed up according to standard procedure in the second year. No consultation is scheduled in advance during the telemonitoring year. Consultations can take place at the request of doctors or patients, if required. An e-mail is sent to investigators once a month to remind them to remotely monitor their patients. Medical alerts are programmed and can be adapted for individual patients and e-mailed to investigating physicians with a link to connect to the monitoring platform. These alerts highlight hypoglycemia with cases of hyperglycemia accounting for over 30% of the blood glucose levels recorded, and excessive weight loss or weight gain.

In this study a hypoglycemia alert is triggered when the patient’s blood glucose level is under 3.3 mmol/l (59.45 mg/dl) and a hyperglycemia alert is triggered when the patient’s blood glucose level is over 8.8 mmol/l (160 mg/dl). However, these thresholds are modular and can be adjusted by the physician according to the patient profile.

These alerts should not be viewed as emergency cases but provide investigators with information on events over the last 15–30 days. There is no procedure to follow the physician judge the therapeutic action to be brought.

The project team receives technical alerts by e-mail. These technical alerts inform the project team if patients have not connected to the system for more than 15 days. In response, they contact the patients via the platform e-mailing system or by telephone to resolve any technical problems provide advice or motivate patients.

##### The secure messaging system

A secure messaging system facilitates patient contact and coaching.

The physician according to the telemonitoring sends messages to patients for coaching.

Patients can choose the receiver of the message depending on the nature of the request (medical or technical).

##### The telemonitoring report

Investigators are asked to provide a telemonitoring report in the form of a pop-up menu. They are required to sign the telemonitoring document and provide an estimate of the amount of time spent on telemonitoring from a medical-economic standpoint.

#### Outcomes

The primary endpoint is the difference between HbA1c level after 1 year of follow-up and the targeted HbA1c level defined at baseline by the physician in charge of the patient (based on the recommended strategy for glycemic control in type 2 diabetes) [17, 18]. HbA1c levels are measured by high-performance liquid chromatographic (HPLC) methods HbA1c levels will be measured at baseline, 3, 6, 9 and 12 months of follow-up.

The secondary outcomes are:

Comparison between the two arms for assessment at baseline and after 1 year of follow-up of:

- Improvement of nutritional knowledge, questionnaires will be given to patients.
- Dietary intake measured by a validated food survey based on 3-day food record.
- Physical activity practice, measured by the general physical activity questionnaire (GPAQ.
- Body composition, BMI (body mass index), waist circumference will be measured.
- Mean of blood glycaemia, self-monitoring blood glucose books will be copied.
- Total cholesterol, triglycerides and high density lipoprotein (HDL) cholesterol blood levels, a blood sample will be collected.
- Quality of life of patients, measured by the diabetes quality of life questionnaire (DQOL) and the EQ-5D questionnaires.

Direct and indirect costs will be recorded at baseline, throughout the study and at the closure visit. The economic analysis will be carried out from the cost payer’s point of view, Assurance Maladie (Health Insurance). Direct medical costs (directly attributable to patient treatment), direct costs of a non-medical nature (travelling expenses and the costs incurred through implementing the telemonitoring programme) and indirect costs (production losses) to be paid by health insurance, mutual insurance and patients will be calculated.

Specifically for the intervention group, assessment of the evolution of parameters collected with the telemonitoring device.

- Food behavior through meals registered with the Nutri-Educ software.
- Nutritional knowledge through rate of good choices playing games of Nutri-Kiosk software.
- Physical activity measures collected with the podometer (number of steps, number of stairs climbed up and intensity).
- Weight, fat rate and lean rate collected with the balance.
- Means of glycaemia collected with the self-monitoring blood glucose device.
- Acceptability and satisfaction of the telemonitoring system by the patients with the number and time of connections during the 1st year of follow-up and a questionnaire at 1 year.
- Acceptability and satisfaction of the telemonitoring system by the health professionals with the number and time of connections leading to a medical decision (phone call or medical appointment) during the 1st year of follow-up and a questionnaire at 1 year.

#### Study duration and schedule

The overall duration of this study is 4.5 years comprising a recruitment period of 2.5 years and a 2-year follow-up period for each patient enrolled. Clinical and economic data are recorded over the 2 year follow-up period.

#### Sample size

The sample size was calculated using the approach proposed by Frison and Pocock [19]. The calculation is based on the comparison between the two study arms of the mean of 4 post-treatment measurements during the 1st year of follow-up (at 3, 6, 9 and 12 months). Assuming a standard deviation of 2 points of HbA1c, a correlation of 0.4 between the pre- and post-randomization measurements, and a correlation of 0.5 between the post-randomization measurements, 117 patients are required per arm (234 patients in all) to detect a difference of 0.5 points of HbA1c with a power of 80%, a two-sided type I error of 5%. This calculation was computed using Stata SE 11.2 software. To avoid any loss of power due to patients lost to follow-up, the cohort obtained is increased by 20% to give a total of 282 patients to be enrolled (141 per arm).

#### Allocation

Randomization was based on a 1:1 ratio and stratified on the baseline HbA1c level (<7.5% vs ≥7.5%). The allocation sequence was randomly permuted in blocks of varying size (2, 4 or 6), generated by computer (Stata SE 11.2, ralloc procedure) by the methodologist. There was no blinding procedure in this open-label trial, however randomization was centralized at the Toulouse University Hospital and managed by the project manager and the two clinical research associates, and the allocation sequence was unknown to the investigators who enrolled participants.

#### Data collection method

Data are collected by the investigators, study staff and by patients called PROs (patient report outcomes) PROs. Hba1c levels, serum lipid profile, clinical exam and questionnaires were recorded on an e-crf (e-clinical report form) by single data entry.

Quarterly, the CRAs (clinical research associate) call the patients, over the 2-year period, to obtain protocol-related information: result of the HbA1c assay, medical treatments and adverse events.

Data obtained using biomedical sensors will be retained. Range checks were implemented to limit data entry mistakes on the e-crf. All the data obtained from the various sources (biomedical sensors, tablet, data entered by the clinicians, data entered by the CRA) will be merged into a single complete database. A simple input will be made on an input mask with logical controls for the clinical data. The data will be validated according to the data management plan defined jointly by the coordinating investigator and the epidemiology department (methodologist and statistician).

The data will be frozen after the last logical and coherence checks carried out at the beginning of the statistical analysis. Once all the CRFs have been entered and validated, and all the steps of the Data Management have been locked, the Data Manager can proceed to freeze the database, a certificate of which will be issued to the Project team.

The database will then be provided to the Statistician for analysis in accordance with the statistical analysis plan.

#### Statistical methods

##### Analysis population

Efficacy will be analyzed on an intent-to-treat (ITT) basis followed by a per protocol (PP) analysis, if required. The PP analysis will only be carried out if the ITT population differs from the PP population by more than 10%. A flow chart will be described and a detailed descriptive analysis of the distribution of baseline variables in the study population will be carried out per arm in order to check the randomization result (intervention/control) and globally.

##### Primary outcome analysis

The primary outcome (difference between HbA1c level after 1 year of follow-up and baseline targeted HbA1c level) will be compared between the two groups. The difference between the the intervention and control groups will be assessed firstly using bivariate analysis (Student’s T test), and secondly using mixed model analysis for repeated measures. The mixed model will be adjusted for the baseline value of HbA1c, as well as additional baseline variables that might be potential confounders: center, age, gender, treatment by insulin, duration of diabetes and history of diabetes complications. To manage missing data, a sensitivity analysis using the last observation carried forward (LOCF) technique will be applied. Secondary outcomes will be analyzed according to the same principles. A bivariate analysis will be computed, followed by a multivariate analysis of covariance (ANCOVA) adjusted for the baseline value of the corresponding secondary outcome and the same set of potential baseline confounders than in the primary outcome analysis.

##### Methods for medical-economic analysis

A cost/efficacy ratio will be established:

Differential RCE = Δ Cost between strategies/Δ Efficacy between strategies.

Markovian modelling can be used for the precise estimation of the various costs incurred by the diabetes treatment strategies and the medical repercussions over the 2-year period including published data [20, 21] and expert advice. A sensitivity analysis will be carried out.

#### Data monitoring

A clinical Research associate mandated by the promotor visits regularly each study site and performs all types of site visits including, but not limited to, qualification visits, initiation visits, monitoring visits and termination visits.

During the site visits the CRA will monitor the informed consent, ensure the trial is conducted in compliance with the protocol, ICH/GCP, applicable regulatory requirements and applicable SOPs/work instructions. The CRA will be responsible with trial monitoring on-site of clinical data for accuracy and completeness, and clinical report preparation and presentation.

#### Declaration of serious adverse event

The investigator evaluates each adverse event in terms of its severity.

The pharmacovigilance unit of the Toulouse University Hospital has established a specific form to be completed by the investigator who declares the SAE (serious adverse event) and sends it back.

He must notify the sponsor, without delay from the day of his knowledge, of any serious adverse event from the date of signature of the consent, throughout the duration of the patient’s follow-up planned by the research and up to 8 days after the completion of the participant’s research follow-up, where it is likely to be due to research,

#### Auditing

An audit can be carried out at any time by persons mandated by the promoter and independent of the researchers. Its objective is to ensure the quality of the research, the validity of its results and the respect of the law and the regulations in force.

## Ethics and dissemination

### Regulatory authorizations

All of the authorizations required in accordance with French legislation were obtained for this study: Agence Nationale de Sécurité du Médicament (ANSM) (authorization dated 10/05/2013), Comité Consultatif sur le Traitement de l’Information en matière de Recherche dans le domaine de la Santé (CCTIRS) (Approval Granted on 22/05/2013), Comité de Protection des Personnes (CPP) Sud-Ouest et Outre-Mer (SOOM) (Approval Granted on 27/05/2013), Commission Nationale Informatique et Libertés (CNIL) (Approval Granted on 22/10/2013). This study has been assigned the following numbers: ClinicalTrials No. NCT01955031 and ID-RCB number: 2013-A00391-44.

## Discussion

We hope that the telemonitoring of patients in their own home will lead to new organizational management methods in response to relevant follow-up (consultations not scheduled in advance and appointments made depending on the patients’ state of health). Furthermore, refocusing targets based on the information recorded should have a beneficial impact on patient knowledge, behavior and blood glucose management, preventing diabetic complications in the longer term [22]. Apart from medication, the management of patients with type 2 diabetes is multi-faceted and includes education to promote greater independence, the monitoring of blood glucose levels and changes in lifestyle. It is not easy to change the lifestyle of patients who are set in their ways or to find out more about their physical activities and daily diet. Similarly, it is not easy to sustain the change of attitude needed to control the disease.

Teleeducation tools and connected health devices for telemonitoring purposes can provide both doctors and patients with objective data to discuss optimum solutions on a case-by-case basis. The connected devices per se can be used to teach patients with the support of coaching [23].

We hope that telemonitoring over several months of patients with type 2 diabetes will provide patients with a better understanding of what they need to do to improve blood glucose management and give them the incentive to continue their efforts in the longer term.

Apart from the primary objective concerning blood glucose management, the results of the secondary objectives concerning weight, diet and quality of life should enable us to set targets which are more patient-specific.

The cost-efficacy study will help to establish the economic model to be considered in order to continue this type of activity.

In conclusion, educating patients at home about a balanced diet and physical exercise could be extended to a bigger diabetic cohort if it proves to be cost-effective. Education concerning diet and the introduction of appropriate physical exercise could be used to educate patients with other chronic cardiovascular diseases, obesity or respiratory diseases, etc.

The telemonitoring of chronic diseases is in its beginning. Other studies should highlight patient-specific telemonitoring tools (suitable patients, when, for how long, combination or individual telemonitoring and teleeducation, delegation of tasks and the role of treating physicians, etc.).

## Abbreviations

ANSM: Agence Nationale de Sécurité du Médicament (French National Agency for the Safety of Medicines)
BMI: body mass index
CCTIRS: Comité Consultatif sur le Traitement de l’Information en matière de Recherche dans le domaine de la Santé (French Advisory Committee on Information Processing in Material Research in the Field of Health)
CNIL: Commission Nationale Informatique et Libertés (French National Commission on Data Processing and Liberty)
CPP: Comité de Protection des Personnes–EC (Ethics Committee)
CRA: clinical research assistant
CRF: clinical report form
DQOL: diabetes quality of life
EQ5D: EuroQol five dimensions questionnaire
GPAQ: global physical activity questionnaire
HAS: Haute Autorité de Santé (French Health Authorities)
HbA1c: glycated hemoglobin
ICH/GCP: International Conference on Harmonisation/Good Clinical Practise
ITT: intent to treat
PP: per protocol
SAE: serious adverse event
SOP: standard operating procedure
WHO: World Health Organization
